# RNA capping by mitochondrial and multi-subunit RNA polymerases

**DOI:** 10.1080/21541264.2018.1456258

**Published:** 2018-04-25

**Authors:** Christina Julius, Amber Riaz-Bradley, Yulia Yuzenkova

**Affiliations:** Centre for Bacterial Cell Biology, Institute for Cell and Molecular Biosciences, Newcastle University, Newcastle upon Tyne, NE2 4AX, UK

**Keywords:** transcription, RNA polymerase, RNA capping, non-canonical capping, NAD^+^, FAD, dephospho coenzymeA, UDP-GlcNAc, mitochondrial RNA polymerase

## Abstract

Recently, it was found that bacterial and eukaryotic transcripts are capped with cellular cofactors installed by their respective RNA polymerases (RNAPs) during transcription initiation. We now show that mitochondrial RNAP efficiently caps transcripts with ADP – containing cofactors. However, a functional role of universal RNAP – catalysed capping is not yet clear.

## The discovery of non-canonical transcript capping

**Figure 1.**
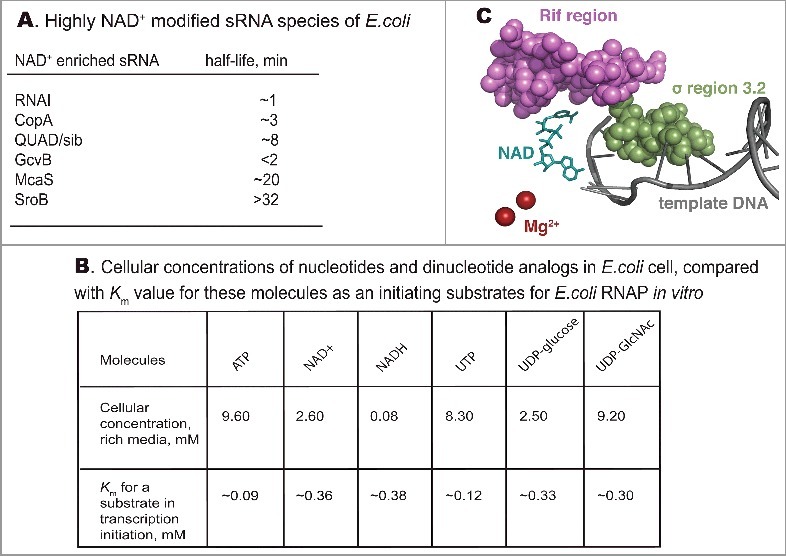
A. A list of 6 heavily NAD^+^ modified RNA species found by Cahova et. al., [1] with half-lives reported in [22]. B. Cellular concentrations of nucleotides and analogs in *E. coli* cell reported by Bennett et. al., [9], and the *K*_m_ for their usage as a substrates in transcription initiation [8]. C. Regions of RNAP shown to influence capping efficiency. PDB 5D4D structure of *Thermus thermophilus* RNAP open complex with NADpC was used, NAD is shown in cyan. Part of rifampicin-binding pocket corresponding to cluster I of Rif region of β subunit is in magenta, region 3.2 of σ subunit is in green, template DNA is grey, Mg^2+^ ions are in ruby.

Capping of RNA is no longer seen as an exclusive feature of eukaryotes, thanks to the recent discovery of bacterial transcripts capped by NAD^+^ and 3′-dephosphocoenzyme A (DP-CoA) [1,2]. NAD^+^ is the only cap investigated *in vivo* in *E. coli*, and is found on a number of small RNAs (sRNAs) and messenger RNAs (mRNAs). In addition, a number of currently uncharacterised moieties were found attached to *E.coli* cellular RNA which could potentially also serve as 5’ RNA caps [3]. The extent of NAD^+^ modification (NADylation) in the cell varies greatly for different RNA species. The RNA species that are most heavily NADylated *in vivo* [1] are listed on Figure 1A. Even for these species, only a relatively small proportion of the transcripts are capped with NAD^+^ (13% in the case of most heavily NADylated species, namely RNAI – the antisense RNA involved in the regulation of pUC19 plasmid replication [1]). More recently NAD^+^ capping was shown not to be unique for bacteria, as NADylated RNAs were found *in vivo* in *Saccharomyces cerevisiae* and human cells [4,5].

The search for an enzyme that can potentially NADylate RNA transcripts was relatively straightforward, as bacterial RNA polymerase (RNAP) was shown previously to use NAD^+^ as an initiating nucleotide (given its ADP moiety and free 3’ hydroxyl group) [6]. Studies by Bird et al., and Julius and Yuzenkova, using promoter-specific assays, demonstrated that capping can be performed by RNAP on promoters where transcription starts with A [7,8]. These studies showed that the *K*_m_ for NAD^+^ in transcription initiation was much lower than the *in vivo* concentration of NAD^+^ (Figure 1B). Furthermore, Bird et al. observed a strong correlation between the extent of NADylation of a chosen transcript *in vivo* and the efficiency of NADylation by RNAP *in vitro* [7]. Eukaryotic RNApol II was also shown to be able to incorporate NAD^+^, suggesting that the NADylated transcripts observed *in vivo* are also capped by RNAP [7].

Other ADP-containing cofactors were shown to be efficiently incorporated at the 5’ end of RNA by RNAP, such as FAD and 3′-dephosphocoenzyme A (but not NADP and NADPH) [7,8]. The efficiency of incorporation for these compounds and their concentration in the cell are lower than those for NAD^+^, suggesting that the possible abundance of these caps is also lower [9].

## Cell wall precursors are potentially another class of prokaryotic capping molecules

Dinucleotides UDP-Glucose and UDP-GlcNAc, the precursors of bacterial cell wall synthesis, are even more abundant than NAD^+^ in *E. coli* cells grown on rich media (Figure 1B). We recently found that for promoters coding for U at position +1, their RNA transcripts can be efficiently capped *in vitro* by *E. coli* RNA polymerase with UDP-GlcNAc and UDP-Glucose [8]. The relatively low *K*_m_ for the incorporation of these substrate at the 5’ end of the RNA transcripts by RNAP, favours the probability of *in vivo* capping by UDP-GlcNAc and UDP-Glucose, by analogy with NAD^+^ (Figure 1B). Although less than 10% of *E. coli* promoters code for U at position +1, a link between gene expression and cell wall synthesis could be of potential significance for coordinating biomass and cell wall synthesis.

The ability of RNAP to incorporate variety of known nucleotide-containing molecules at the 5’ position of transcript, as well as a number of identified but uncharacterised RNA modifying moieties [3], suggests the existence of a wide repertoire of RNA caps in the cell.

## At least two domains of bacterial RNAP determine efficiency of NAD^+^ capping

We showed that initiation with NAD^+^ stabilises short transcripts and favours promoter escape by *E. coli* RNAP *in vitro* [8]. Whether this stabilisation comes via additional base pairing of cap with the -1 position of the promoter (since NAD^+^ has a nicotine mononucleotide moiety, which may potentially interact with DNA template at -1 position) remains somewhat controversial. Bird et al. showed that the identity of the base at position -1 (-1A vs -1C) affects the efficiency of capping [7]. However, our data suggests that the base at -1 affects initiation in general, without changing the preference for NAD^+^ [8]. Indeed, in the crystal structure of the RNAP initiation complex with a short NADylated transcript, the NMN moiety does not make contacts with DNA but rather faces the protein [7] (Figure 1C). Also, in agreement with the crystal structure, we showed that amino acid changes in the rifampicin-binding pocket of RNAP strongly affected the efficiency of NAD^+^ incorporation, suggesting that observed stabilisation of short capped RNAs is due to interactions between the NAD^+^ cap and the RNAP rifampicin-binding pocket [8] (Figure 1C). Therefore, different configuration of rifampicin-binding pocket may affect NADylation of RNA capping in different bacteria. In contrast to NAD^+^, the incorporation of UDP-containing cell wall precursors was not affected by the amino acid substitutions in the rifampicin-binding pocket.

Cofactors bound at +1 position may potentially interact with the 3.2 region of initiation factor σ^70^, which protrudes towards the RNAP active centre [10]. However, we found that a mutant version of σ^70^ lacking region 3.2 (σ^70Δ3.2^) had no effect on incorporation of NAD^+^, NADH, FAD, UDP-Glucose or UDP-GlcNAc. In contrast, the mature cell wall precursor UDP-MurNAc-pentapeptide, was incorporated by the σ ^70Δ3.2^ mutant of RNAP much more efficiently, suggesting that region 3.2 of σ^70^ may serve to prevent incorporation of advanced cell wall precursors [8] (Figure 1C). Region 3.2 is absent from many sigma factors, suggesting that alternative sigma subunits may allow capping with bulky substrates.

## Decapping enzymes for non-canonical caps

The discovery of NudC (NUDIX nicotinamide pyrophosphohydrolase) as an enzyme that removes the NAD^+^ cap from RNA made the parallel between classic eukaryotic and non-canonical capping processes even more striking. *E. coli* NudC was initially described as a housecleaning enzyme hydrolyzing the pyrophosphate bond of NAD^+^/NADH to produce nicotinamide mononucleotide (NMN^+^/NMNH) and AMP [11]. Recently, it was shown than NudC efficiently removes the NAD^+^/NADH cap to produces 5′-monophosphorylated RNA [1]. In eukaryotes, the role of NudC in decapping could be played by NUDIX hydrolases NPY1 in *Saccharomyces cerevisiae* and Nudt19 in *Oryza sativa*, which both showed decapping activity *in vitro* [12]. The activity spectrum of the bacterial NudC is relatively wide; it can remove several ADP analogues from RNA *in vitro*, including DP-CoA [7], consistent with its hydrolase activity towards a broad range of dinucleotides [11].

Removal of cap by NudC was proposed to be the first stage in the degradation of capped RNA to produce a monophosphorylated species, which are a preferred substrate for endonuclease RNaseE [13]. Curiously, however, NudC was not associated with McaS (IsrA) sRNA [14], one of the most highly NADylated sRNAs in *E. coli* (Figure 1A), while other known components of the RNA degradation machinery, such as RNaseE, RNA helicase RhlE and PNPase, were present [14]. This may suggest that the involvement NudC in RNA maturation might be more complex. Differential decapping by NudC, and its association with target RNAs, could be influenced by the secondary structures of RNAs, as NudC is single-strand dependent [13].

NudC is orthologous to the RppH NUDIX hydrolase, which removes pyrophosphate from triphosporylated RNA (leaving 5’ monophosphate) [15]. A number of additional poorly characterised NUDIX hydrolases in *E. coli* [16] suggests that there might be more potential decapping enzymes for different caps. Notably, neither NudC nor RppH are essential for *E. coli* under normal growth conditions, suggesting possible redundancy of the decapping activities.

## Human mitochondrial RNAP efficiently caps RNA with NAD^+^ and other ADP-containing cofactors

**Figure 2.**
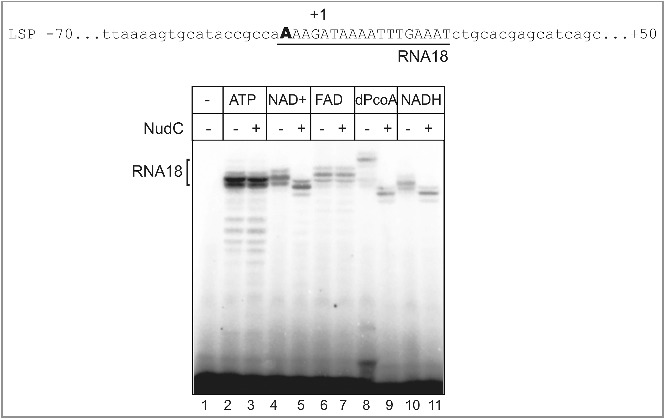
Human mitochondrial RNAP (hmRNAP) incorporates ADP analogs *in vitro*. A partial sequence of the light strand promoter (LSP) is shown, with the initially transcribed sequence underlined. For the assay, 50 nM TFAM, 50 nM hmRNAP, 50 nM TFB2M (purified as described in [23]) were combined with 50 nM of linear DNA fragment containing LSP promoter (positions -70 to +50) in 10 µl of transcription buffer (40 mM Tris pH 8.0, 10 mM MgCl_2_, 10 mM DTT), then ATP or ADP analogs were added to the final concentration of 1mM. Transcription was initiated by the addition of 10 mM MgCl_2_, 50 µM ATP, 300 µM GTP, 10µM [α^32^P]-UTP, 25 Ci/mmol (Hartmann Analytic). After 30 min incubation at 37°C, 500 nM NudC was added to half of the reactions and incubated for additional 15 minutes at 37°C. Transcripts modified with NAD^+^, NADH and DP-CoA (but not ATP or FAD) were susceptible to NudC (lanes 5,9,11) judging from increased mobility of the products. Reactions were stopped by the addition of formamide-containing loading buffer. Products were separated on denaturing polyacrylamide gels (20% acrylamide, 3% bis-acrylamide, 6M urea, 1xTBE), revealed by PhosphorImaging (GE Healthcare), and analysed using ImageQuant software (GE Healthcare).

Recently, mitochondrial transcripts capped with NAD^+^ were detected in human cells [5]. Mitochondria contain a major cellular pool of NAD^+^ (up to 70%), where it is used for redox reactions and for signalling [17]. We explored the possibility that mitochondrial RNAP (mtRNAP) could cap RNA via a mechanism that is similar to multi-subunit RNAPs. We found that human mtRNAP (hmRNAP) efficiently initiates transcription with NAD^+^, NADH, FAD and DP-CoA on the light strand promoter (LSP; one of the only two human mitochondrial promoters) *in vitro* (Figure 2). The efficiency of initiation with NAD^+^ was approximately 25% compared to ATP, while the other cofactors showed of between 10 to 15%. Our results suggest that mtRNAP is likely to be responsible for adding a NAD^+^ cap to mitochondrial transcripts. Capping in human mitochondria might have consequences for both translation and replication in these organelles. The initially transcribed sequences from both mitochondrial promoters are precursors of tRNAs. It is therefore possible that 5’ NADylation might affect their maturation process. Additionally, RNA synthesised from the LSP promoter serves as replication primer [18], and its capping might influence initiation of replication, primer removal and subsequent DNA ligation.

## Emerging physiological roles of non-canonical capping

The first experimentally confirmed role for non-canonical NAD^+^ cap in bacteria is an increased resistance to degradation, shown for RNAI in the absence of NudC processing [7]. However, this remains controversial, since in other studies [1] deletion of NudC did not affect the overall stability of the RNAI and GcvB populations, the two RNAs most heavily NADylated *in vivo.* Moreover, overall stability of NADylated sRNAs varies widely in wild type *E. coli*, and there is no direct correlation between NADylation and stability (Figure 1A). Notably, in contrast to *E. coli*, NADylation in eukaryotes promotes mRNA decay [5] via decapping by the DXO enzyme, which might additionally supply its 5′-3′ degradation activity.

The existence of subpopulations of capped RNA may play a potential role in bistability, the creation of phenotypic variability among clonal population that bacteria use in processes such as dormancy, persistence and sporulation [19]. Capping with dinucleotide analogues might play role in number of regulatory processes involving unstable regulatory RNAs. One example of such process is the type I toxin-antitoxin systems in bacteria, based on translational repression of toxin mRNA by an antisense RNA. This idea is supported by high *in vivo* NADylation of QUAD (sib) RNA (Figure 1A) – antitoxin sRNA preventing the production of the small protein that depolarises the cellular membrane [20].

The extent of capping could be responsive to the changes in cellular metabolism. For example, in *E.coli*, the proportion of NAD^+^ capped RNAI found in stationary phase compared to exponential phase was two-fold higher [1]. Similarly, in yeast, more capped RNA was found in cells grown on synthetic media compared to those grown on the rich media [4]. Since the NAD^+^/NADH balance plays key role in cellular redox homeostasis, capping could connect transcription directly to the cell's redox state. Given the affinity for NAD^+^ is roughly the same as for NADH (Figure 1B), changes in their cellular concentrations will be directly mirrored by the capping of RNA with NAD^+^ or NADH. The functioning of such signalling of course would depend on a mechanism recognising NADylated from NADHylated RNAs. UDP-Glucose and UDP-GlcNAc are the initial substrates for the cascade of reactions leading to the synthesis of cell wall components. It would be tempting to speculate that expression of some cell wall synthesising enzymes could be controlled directly by the pool of UDP-GlcNAc via capping of +1U transcripts. In general, being rare, RNA modification with cell wall precursors might provide a better regulatory potential, compared to ubiquitous capping with ADP analogs.

Capping might potentially influence translation initiation on a leaderless mRNA.

Another potential cellular role of capping could be the targeting of a specific RNA species, via its cofactor cap, to a protein with affinity for the cognate cofactor, or to a specific subcellular location, *e.g.* to the vicinity of the membrane in the case of UDP-GlcNAc capped RNA.

Intriguingly, we showed that a number of rifampicin resistant RNAPs, including the most widespread clinical isolates, are deficient in capping [8]. This deficiency may contribute to the overall fitness reduction, characteristic for a rifampicin resistant strains [21].

To conclude, despite recent progress, the understanding of non-canonical RNA capping by RNAPs is still patchy*.* More information is needed to put this type of RNA 5’ modification into the category of functional capping, rather than a side reaction of RNAPs. Currently it is hard to envisage a “classic” regulation by the stochastic process of capping. Nevertheless, this “unavoidable” side reaction has to be either used to some advantage or, alternatively, fought against. Both scenarios would have wide ranging cellular consequences with multiple regulatory mechanisms involved. It seems that RNAP has a limited ability to control capping process, apart from alternative sigma factors exchange. It is more feasible to regulate amounts of capped RNA post-transcriptionally, by the linked processes of decapping, alternative folding and RNA chaperons binding. Being a stochastic process, capping might generate variability in a clonal population, which can be exploited at a population level to benefit the organism in adaptation to rapid change in growth conditions. At present, an exact roles of various non-canonical caps in bacteria, eukaryotes and mitochondria are still to be established, as well as full repertoire of enzymes that process non-canonically capped RNAs are to be characterised.

## References

[1] Cahova H, Winz ML, Hofer K, et al. NAD captureSeq indicates NAD as a bacterial cap for a subset of regulatory RNAs. Nature. 2015;519(7543):374–377. doi:10.1038/nature14020. PMID:25533955 25533955

[2] Kowtoniuk WE, Shen Y, Heemstra JM, et al. A chemical screen for biological small molecule-RNA conjugates reveals CoA-linked RNA. Proc Natl Acad Sci U S A. 2009;106(19):7768–7773. doi:10.1073/pnas.0900528106. PMID:19416889; PMCID:. 19416889 PMC2674394

[3] Chen YG, Kowtoniuk WE, Agarwal I, et al. LC/MS analysis of cellular RNA reveals NAD-linked RNA. Nat Chem Biol. 2009;5(12):879–881. doi:10.1038/nchembio.235. PMID:19820715; PMCID:. 19820715 PMC2842606

[4] Walters RW, Matheny T, Mizoue LS, et al. Identification of NAD+ capped mRNAs in Saccharomyces cerevisiae. Proc Natl Acad Sci U S A. 2017;114(3):480–485. doi:10.1073/pnas.1619369114. PMID:28031484; PMCID:28031484 PMC5255579

[5] Jiao X, Doamekpor SK, Bird JG, et al. 5' End Nicotinamide Adenine Dinucleotide cap in human cells promotes RNA decay through DXO-mediated deNADding. Cell. 2017;168(6):1015–1027 e10. doi:10.1016/j.cell.2017.02.019. PMID:28283058; PMCID:28283058 PMC5371429

[6] Malygin AG, Shemyakin MF Adenosine, NAD and FAD can initiate template-dependent RNA synthesis catalyzed by Escherichia coli RNA polymerase. FEBS Lett. 1979;102(1):51–54. doi:10.1016/0014-5793(79)80926-6. PMID:222618 222618

[7] Bird JG, Zhang Y, Tian Y, et al. The mechanism of RNA 5' capping with NAD+, NADH and desphospho-CoA. Nature. 2016;535(7612):444–447. doi:10.1038/nature18622. PMID:27383794; PMCID:27383794 PMC4961592

[8] Julius C, Yuzenkova Y Bacterial RNA polymerase caps RNA with various cofactors and cell wall precursors. Nucleic Acids Res. 2017;45(14):8282–8290. doi:10.1093/nar/gkx452. PMID:28531287; PMCID:28531287 PMC5737558

[9] Bennett BD, Kimball EH, Gao M, et al. Absolute metabolite concentrations and implied enzyme active site occupancy in Escherichia coli. Nat Chem Biol. 2009;5(8):593–599. doi:10.1038/nchembio.186. PMID:19561621; PMCID:19561621 PMC2754216

[10] Kulbachinskiy A, Mustaev A Region 3.2 of the sigma subunit contributes to the binding of the 3'-initiating nucleotide in the RNA polymerase active center and facilitates promoter clearance during initiation. J Biol Chem. 2006;281(27):18273–18276. doi:10.1074/jbc.C600060200. PMID:16690607 16690607

[11] Frick DN, Bessman MJ Cloning, purification, and properties of a novel NADH pyrophosphatase. Evidence for a nucleotide pyrophosphatase catalytic domain in MutT-like enzymes. J Biol Chem. 1995;270(4):1529–1534. doi:10.1074/jbc.270.4.1529. PMID:7829480 7829480

[12] Zhang D, Liu Y, Wang Q, et al. Structural basis of prokaryotic NAD-RNA decapping by NudC. Cell Res. 2016;26(9):1062–1066. doi:10.1038/cr.2016.98. PMID:27561816; PMCID:27561816 PMC5034116

[13] Hofer K, Li S, Abele F, et al. Structure and function of the bacterial decapping enzyme NudC. Nat Chem Biol. 2016;12(9):730–734. doi:10.1038/nchembio.2132. PMID:27428510; PMCID:27428510 PMC5003112

[14] van Nues RW, Castro-Roa D, Yuzenkova Y, et al. Ribonucleoprotein particles of bacterial small non-coding RNA IsrA (IS61 or McaS) and its interaction with RNA polymerase core may link transcription to mRNA fate. Nucleic Acids Res. 2016;44(6):2577–2592. doi:10.1093/nar/gkv1302. PMID:26609136; PMCID:26609136 PMC4824073

[15] Deana A, Celesnik H, Belasco JG The bacterial enzyme RppH triggers messenger RNA degradation by 5' pyrophosphate removal. Nature. 2008;451(7176):355–358. doi:10.1038/nature06475. PMID:18202662 18202662

[16] McLennan AG The Nudix hydrolase superfamily. Cell Mol Life Sci. 2006;63(2):123–143. doi:10.1007/s00018-005-5386-7. PMID:16378245 16378245 PMC11136074

[17] Stein LR, Imai S The dynamic regulation of NAD metabolism in mitochondria. Trends Endocrinol Metabol. 2012;23(9):420–428. doi:10.1016/j.tem.2012.06.005. PMID:22819213; PMCID:22819213 PMC3683958

[18] Wanrooij PH, Uhler JP, Simonsson T, et al. G-quadruplex structures in RNA stimulate mitochondrial transcription termination and primer formation. Proc Natl Acad Sci U S A. 2010;107(37):16072–16077. doi:10.1073/pnas.1006026107. PMID:20798345; PMCID:20798345 PMC2941323

[19] Veening JW, Stewart EJ, Berngruber TW, et al. Bet-hedging and epigenetic inheritance in bacterial cell development. Proc Natl Acad Sci U S A. 2008;105(11):4393–4398. doi:10.1073/pnas.0700463105. PMID:18326026; PMCID:. 18326026 PMC2393751

[20] Fozo EM. New type I toxin-antitoxin families from “wild” and laboratory strains of E. coli: Ibs-Sib, ShoB-OhsC and Zor-Orz. RNA Biol. 2012;9(12):1504–1512. doi:10.4161/rna.22568. PMID:23182878 23182878

[21] Melnyk AH, Wong A, Kassen R The fitness costs of antibiotic resistance mutations. Evol Appl. 2015;8(3):273–283. doi:10.1111/eva.12196. PMID:25861385; PMCID:25861385 PMC4380921

[22] Chen H, Shiroguchi K, Ge H, et al. Genome-wide study of mRNA degradation and transcript elongation in Escherichia coli. Mol Syst Biol. 2015;11(5):808. doi:10.15252/msb.20159000. PMID:25964259; PMCID:25964259 PMC4461401

[23] Gaspari M, Falkenberg M, Larsson NG, et al. The mitochondrial RNA polymerase contributes critically to promoter specificity in mammalian cells. EMBO J. 2004;23(23):4606–4614. doi:10.1038/sj.emboj.7600465. PMID:15526033; PMCID:15526033 PMC533051

